# Impact of *UGT*1*A1* polymorphisms on Raltegravir and its glucuronide plasma concentrations in a cohort of HIV-1 infected patients

**DOI:** 10.1038/s41598-018-25803-z

**Published:** 2018-05-09

**Authors:** Leïla Belkhir, Carole Seguin-Devaux, Laure Elens, Caroline Pauly, Nicolas Gengler, Serge Schneider, Jean Ruelle, Vincent Haufroid, Bernard Vandercam

**Affiliations:** 1 AIDS Reference center, Cliniques Universitaires Saint-Luc, 1200 Brussels, Belgium; 2 Louvain centre for Toxicology and Applied Pharmacology (LTAP), Institut de recherche expérimentale et clinique (IREC), Université catholique de Louvain (UCL), 1200 Brussels, Belgium; 3 Department of Infection and Immunity, Luxembourg Institute of Health, Esch-sur-Alzette, Luxembourg; 4 Louvain Drug Research institute, UCL, 1200 Brussels, Belgium; 5 Department of toxicology, Laboratoire National de Santé, 3555 Dudelange, Luxembourg; 6 AIDS reference laboratory, IREC, UCL, 1200 Brussels, Belgium; 7 Department of Clinical Chemistry, Cliniques Universitaires Saint-Luc, 1200 Brussels, Belgium

## Abstract

The aim of this study was to evaluate the effect of UGT1A1 polymorphisms on Raltegravir (RAL) and its metabolite RAL-glucuronide trough plasma concentrations ([RAL]plasma and [RAL-glu]plasma) and on the metabolic ratio (MR): [RAL-glu]plasma/[RAL]plasma. UGT1A1 genotyping was performed on 96 patients. 44% (n = 42) were homozygous UGT1A1*1/*1 while 50% (n = 48) and 6% (n = 6) were UGT1A1*28 and UGT1A1*36 carriers, respectively. The median concentration and interquartile range (IQR) of [RAL]plasma were 88.5 ng/ml (41.0–236), 168 ng/ml (85.8–318) and 92.5 ng/ml (36.4–316) for UGT1A1*1/*1, UGT1A1*28 and UGT1A1*36 carriers, respectively. Only the difference between UGT1A1*1/*1 and *28 carriers was statistically significant (p = 0.022). The median MR (IQR) were 5.8 (3–10), 2.9 (1.6–5.3) and 3.2 (1.7–5.9) for UGT1A1*1/*1, UGT1A1*28 and UGT1A1*36 carriers, respectively. Only the difference between UGT1A1*1/*1 and *28 carriers was statistically significant (p = 0.004) with an allele-dependent effect: UGT1A1*28 homozygous having lower MR than heterozygous carriers who show lower MR compared to *1/*1. Except for the sensation of fatigue, this PK effect did not correlate with clinical adverse events or biological abnormalities. In Conclusion, we demonstrate that UGT1A1*28 polymorphism has a significant impact on RAL metabolism: UGT1A1*28 carriers being characterized by higher [RAL]plasma and lower MR.

## Introduction

Raltegravir (RAL) is the first approved drug of the human immunodeficiency virus (HIV)-1 integrase strand inhibitors (INI or INSTI), a class of antiretroviral (ARV) agents. These drugs act by inhibiting the integrase, an HIV-1 specific enzyme which catalyzes the insertion of a DNA copy of the viral genome into the host cell genome^1^. Commonly, RAL is used as a component of antiretroviral therapy (ART) for treatment- naïve or experienced HIV-1 infected patients at a dosage of 400 mg BID.

Initially, RAL was licensed only for treatment-experienced patients on the basis of clinical efficacy and safety data collected in BENCHMRK 1 and 2 trials. These double-blind, randomized studies compared, in patients with therapeutic failure, the virological response between RAL (400 mg BID) and a placebo with both treatments arms receiving an optimized background therapy (OBT). Superior and sustained viral suppression was observed up to 96 weeks with complete virological suppression observed in 57% of the RAL treated group compared to the 27% in the placebo group (p < 0.001)^2^.

Later, in the STARTMRK study, the efficacy of RAL was demonstrated in treatment-naïve patients who reached a sustained virological suppression at least equivalent to Efavirenz (EFV 600 mg QD) up to 156 weeks after initiation of therapy^3^.

Although a first large multicenter study reported more than 10% of patients in clinical practice who develop one drug related-central nervous system (CNS) symptom under RAL treatment^4^, it is now generally considered that RAL has a safe profile with less drug-drug interactions and few clinical adverse events (AE)^2,3^ than observed with other anti-HIV drugs and other integrase inhibitors^5^. Contrarily to most of other anti-HIV drugs, RAL is neither a substrate, nor an inhibitor/inducer of cytochrome P450 (CYP), explaining its moderate drug interaction profile. RAL is a P-glycoprotein (P-gp) substrate but is not described as an inhibitor^6^. The primary metabolic pathway of RAL is a glucuronoconjugation that involves primarily uridine diphosphate glucuronosyltransferase (UGT)1A1 enzyme^7^. The glucuronide metabolite (RAL-glu) is devoid of antiretroviral activity^8^.

UGT1A1 is expressed in the liver and gastrointestinal tract. Its activity is essential in the metabolism of bilirubin^9^. To date, more than 100 variants have been reported in *UGT1A1* gene^10^. Some have been associated either with a decrease (*e.g*. UGT1A1*28, UGT1A1*6) or with an increase (e.g. UGTA1*36) in the UGT1A1 metabolic function. The most thoroughly studied variant of *UGT1A1* is termed as UGT1A1*28 (rs8175347) and is associated with Gilbert’s syndrome. This variant corresponds to a seven thymine–adenine (TA)_7_ dinucleotide repeat in the TATA box at the promoter region of the *UGT1A1* gene as opposed to six (TA_6_) that characterize the wild-type allele (UGT1A1*1)^11^. The distribution of the UGT1A1*28 allele varies across the globe with a minor allelic frequency (MAF) of 26–31% in Caucasians, 42–56% in African–Americans and only 9–16% in Asian populations^12,13^.

UGT1A1*28 variant decreases the activity of UGT1A1 by 25 and 70% depending on the presence of one or two UGT1A1*28 variant allele, respectively^10^. Gilbert’s syndrome is characterized by a chronic mild unconjugated hyperbilirubinemia with a normal liver function due to a 30% residual UGT1A1 activity. The UGT1A1*28/*28 is the most common genotype associated with Gilbert’s syndrome within Caucasian and African populations whereas the UGT1A1*6/*6 (rs4148323, 211 G > A) genotype, is almost exclusively encountered in the Asian populations with a MAF for the UGT1A1*6 allele around 13–16%^14^.

In contrast to these defective alleles, the UGT1A1*36 variant characterized by a 5 TA dinucleotide repeats (TA)_5_ is associated with an increase in UGT1A1 activity and is almost exclusively encountered in the African population with MAF estimated at 3–10%^15^.

Surprisingly, a very limited number of studies have assessed the potential impact of these *UGT1A1* polymorphisms on RAL metabolism and/or plasma concentrations^14,16–19^.

Given the high prevalence of the UGT1A1*28 polymorphism in Caucasian and African populations and its correlation with decreased UGT1A1 activity, this study was conducted to assess the impact of UGT1A1*28 and *36 polymorphisms on RAL metabolism *in vivo* and occurrence of adverse events (AE) in a cohort of mainly Caucasian and African HIV-1 infected patients.

## Materials and Methods

104 HIV-1 infected patients all over 18 years old treated with RAL-containing regimens and followed at the AIDS Reference center of Cliniques Universitaires Saint-Luc in Brussels, Belgium were recruited between November 2012 and June 2015.

In addition to the samples routinely collected (i.e. viral load, CD4-cell count), two more blood samples were drawn immediately prior to pill intake. In order to obtain a post-intake delay as close as possible to the trough sampling time, each patient was personally contacted by phone two days before the study visit to ensure not taking the medication prior to blood sampling.

These two additional samples were used for further determination of both RAL and RAL-glu plasma through concentration ([RAL]_plasma_ and [RAL-glu]_plasma_) and for genomic DNA isolation, respectively.

Plasma samples were isolated by centrifugation at 1125x*g* for 10 min from heparinized blood samples and stored at −20 °C until the day of quantification. The [RAL]_plasma_ and [RAL-glu]_plasma_ were determined using high-pressure liquid chromatography with tandem mass spectrometry (LC-MS/MS) at the Laboratoire National de Santé (LNS), Luxembourg according to a method previously described^20,21^ using Xevo^TM^ TQ MS (Waters, Zellik, Belgium). The instrument was coupled to an ultra-high pressure liquid chromatography system (Acquity UPLC, Waters, Zellik, Belgium). The chromatographic separation was performed on a BEH (bridged ethyl hybrid) C18 1.7 μm column (2.1 × 100 mm) (Waters, Zellik, Belgium). Deuteriated RAL and RAL-glu were used as internal standards. The calibration range for both compounds was 0–160 µg/L. The calculated limits of detection (LOD) were 44 ng/L for RAL and 1.3 ng/L for RAL-glu. Calculated limits of quantitation were 3.3 times LOD. Intraday precision and accuracy were within accepted method validation limits (<15%). The metabolite ratio (MR) of [RAL-glu]_plasma_ on [RAL]_plasma_ was subsequently calculated.

The second blood sample was drawn in an EDTA tube and stored at −20 °C until the day of genotyping analysis. Genomic DNA was extracted from whole blood using a QIAamp® DNA Mini Kit ^TM^ (Qiagen, CA, USA). Identification of UGT1A1*28 allele was performed at Cliniques Universitaires Saint-Luc, Belgium, by using high resolution melting (HRM) curve analysis on a LightCycler 480® (Roche diagnostics) according to a previously described method^22^. The HRM profiles other than 6/6, 6/7 and 7/7 (defined by the number of TA repetition) were subsequently analyzed by direct Sanger sequencing (identification of UGT1A1*36 allele, 5 TA repeats). This genotyping method gave excellent results in the external quality control (EQC) organized by the Reference Institute for Bioanalytics (RfB, Bonn, Germany).

This study protocol (NCT02514369) was approved by the Ethical Committee of UCL Saint-Luc (national number:B403, approval: B403201214460) and all methods were performed in accordance with the relevant guidelines and regulations. Before inclusion, all patients provided their written informed consent to participate in the study.

The statistical analysis was performed using JMP Pro 12 version 12.0.1 for MAC (SAS Institute Inc., Cary, NC, USA).

[RAL]_plasma_ and [RAL-glu]_plasma_ are expressed as median and interquartile range (IQR). Genotype and allele frequencies were calculated and deviations from Hardy-Weinberg equilibrium (HWE) were evaluated using Fisher exact tests. Patients were classified into 3 groups based on the *UGT1A1* allelic status: wild-type (WT, UGTA1*1/*1), UGTA1*28 carriers and UGT1A1*36 carriers.

The differences between the median values of [RAL]_plasma_ and MR among *UGT1A1* allelic status-based groups were analyzed using the Kruskal-Wallis test. Significant results were further analyzed using Steel–Dwass *post-hoc* test. In addition to *post-hoc* analysis, an *a priori* polynomial linear contrast test was performed to assess any potential allele-dependent effect. *P-value* < 0.05 was considered as statistically significant.

## Results

In total, 104 patients were recruited. Among those, 5 patients were excluded due to non-compliance and *UGT1A1* genotyping was performed in 96 patients.

The main clinical characteristics of these 96 remaining patients are reported in Table 1. On average, patients were 52.6 ± 11.7 years old and treated for 42.1 ± 23.7 months. 59 (61%) patients were Caucasian, 34 (35%) were African, two patients were Asian and 1 was South American. 90 (94%) patients had an undetectable viral load (<40 cps/ml) at time of sampling and, among the remaining 6 patients, the median plasma HIV-RNA level was at 69 cps/ml [min-max: 41–152]. The median CD4 cell count was 620 cells/μl [min-max: 88–1418].

**Table 1:** Main characteristics of the patients at day of inclusion.

Number of patients included	96
Age, years (mean ± SD)	52.6 ± 11.7
Body Mass Index, kg/m2 (mean ± SD)	25.8 ± 4.4
Gender, n (%) male	56 (58%)
Ethnic origin, n (%)	
Caucasian	59 (61%)
African	34 (35%)
Asian	2
South American	1
Co-administered ARV drugs	
NRTI	ABC/3TC (n = 8), FTC/TDF (=38), 3TC (n = 17), TDF245mg (n = 8), ABC 600 (n = 1), NRTI-free (n = 25)
NNRTI	ETR (n = 17), NVP (n = 3), EFV/FTC/TDF (n = 2)
PI	DRV (n = 39), ATV (n = 3), LPV/r (n = 3)
MVC	n = 13
CD4 cell count, cells per μl (median [min-max])	620 [88–1418]
Nadir CD4 cell count, cells per μl (median [min-max])	119 [2–402]
HIV-1 RNA < 40 copies per mL, n (%)	90 (94%)
HIV-1 RNA > 40 copies per mL, n (%) copies per mL (median [min-max])	6 (6%) 69 [41–152]
Duration of treatment, months (mean ± SD)	42.1 ± 23.7
Post-intake delay, hours (mean [CI95%])	15 [14.2–15.8]

ARV: antiretroviral, NNRTI: Non-Nucleoside Reverse Transcriptase Inhibitor, NRTI: Nucleoside Reverse Transcriptase Inhibitor, PI: protease inhibitor, DRV: darunavir, ATV: atazanavir, LPV/r: Lopinavir/ritonavir, MVC: maraviroc ETR: etravirine, NVP: nevirapine, ABC: abacavir, 3TC: lamivudine, FTC: emtricitabine, TDF: tenofovir disoproxil fumarate, NRTI-free: without any NRTI.

**Figure 1:**
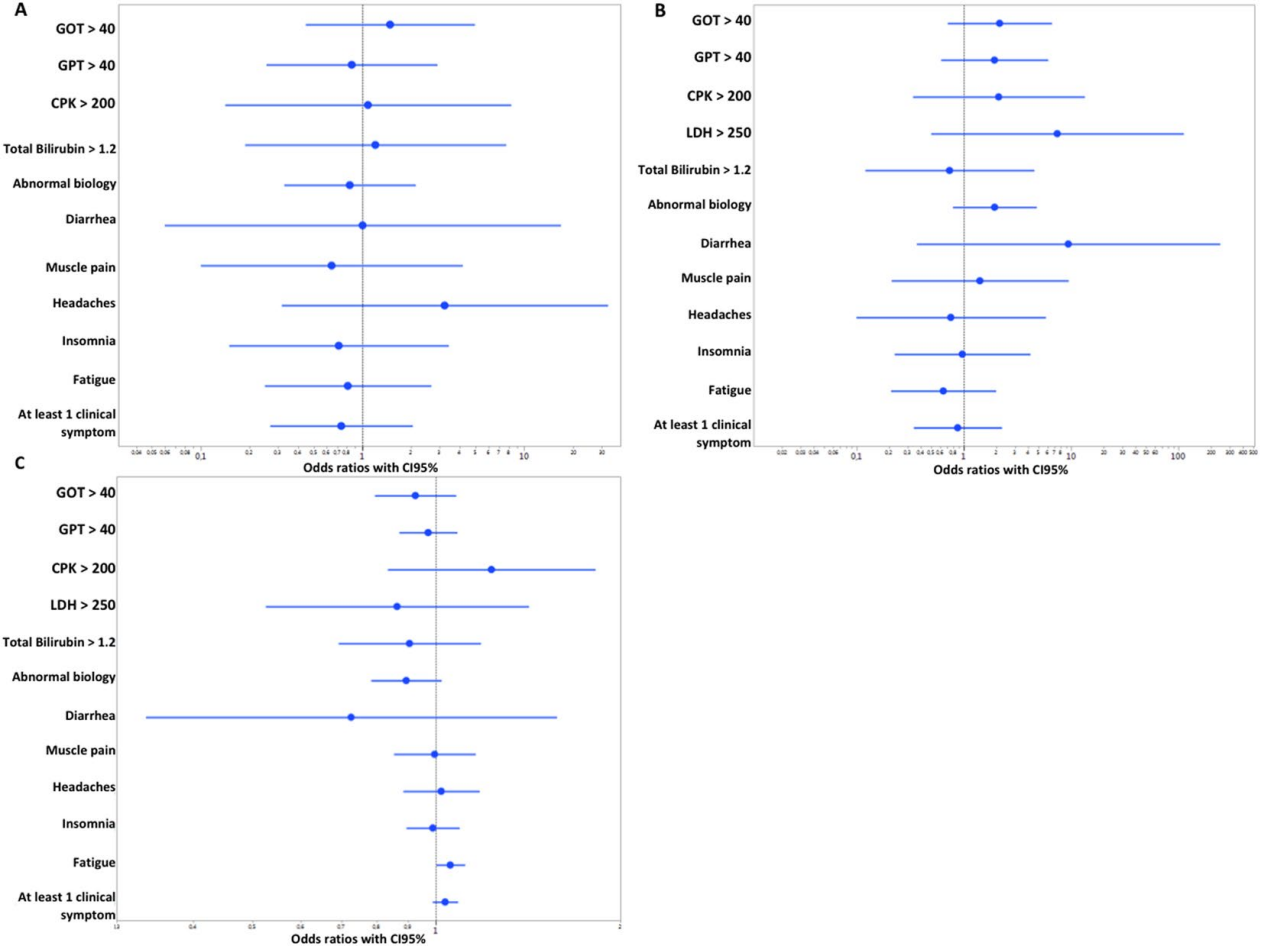
Forest plots displaying odds ratios (OR) with CI_95%_ for the risk of adverse drug reaction associated with (**A**) UGT1A1*28 allele carriership (**B**) an increment of 1 log_10_ unit in plasma RAL concentrations, (**C**) an increment of 1 unit in the MR.

As shown in Table 2, RAL was relatively well tolerated with few clinical adverse events or biological abnormalities. Using a logistic regression test, we did not find any statistically significant association between *UGT1A1* polymorphisms, [RAL]_plasma_ or the [RAL-glu]_plasma_/[RAL]_plasma_ ratio and clinical AE or biological abnormalities except for the sensation of fatigue associated with increased MR value (p = 0,048, likelihood ratio = 3.91, OR = 1.05, Fig. 1).

**Table 2:** Clinical adverse events and biological abnormalities reported in our cohort of 96 patients.

	Number of patients (%)
Clinical adverse events	
Diarrhea	3 (3%)
Muscular pain	3 (3%)
Headache	4 (4%)
Fatigue	13 (13.5%)
Dizziness	1 (1%)
Insomnia	7 (7%)
Biological Abnormalities*	
Total serum bilirubin	
Grade 1 (>ULN − 1.5 × ULN)	4 (4%)
Grade 2 (>1.5–3.0 × ULN)	1 (1%)
CPK	
Grade 1 (>ULN − 2.5 × ULN)	4 (4%)
Grade 2 (>2.5–5 × ULN)	1 (1%)

^*^Grade classification according to “Common Terminology Criteria for Adverse Events v4.0”^34^ CPK: creatinine phosphokinase, ULN: upper limit of normal range.

Concerning the *UGT1A1* polymorphisms, allelic frequencies of UGT1A1*28 and UGT1A1*36 were 35 and 8%, respectively. 44% of patients (n = 42) were homozygous UGT1A1*1/*1 while 50% (n = 48) were UGT1A1*28 carriers (among which 15 homozygous) and 6% (n = 6) were UGT1A1*36 carriers (among which 2 homozygous). The UGT1A1*28 genotype distribution was conformed with the Hardy-Weinberg equilibrium (HWE) in the whole cohort (p = 0.07) as well as in the sub-group of patients from Caucasian and African origin (p = 0.79 and 0.22, respectively). The UGT1A1*36 variant is almost exclusively encountered in the African population and the distribution of UGT1A1*36 genotype was also in agreement with HWE in this population (p = 0.17).

Overall, the median values (IQR) were 131 ng/ml (62.7–303), 468 ng/ml (221–903) and 3.8 (2.1–7) for [RAL]_plasma_, [RAL-glu]_plasma_ and [RAL-glu]_plasma_/[RAL]_plasma_ ratio, respectively.

**Figure 2:**
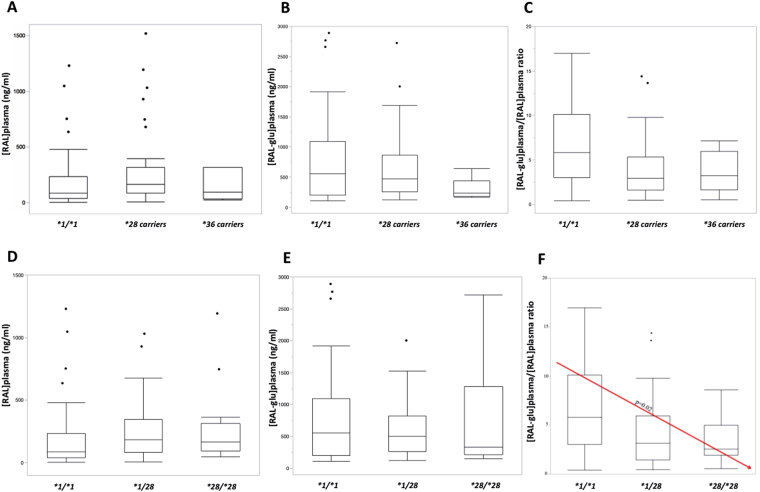
[RAL]plasma (**A**), [RAL-glu]plasma (**B**) and [RAL-glu]plasma/[RAL]plasma ratio (**C**) according to UGT1A1*1/*1, UGT1A1*28 carriers and UGT1A1*36 carriers. [RAL]plasma (**D**), [RAL-glu]plasma (**E**) and [RAL-glu]plasma/[RAL]plasma ratio (**F**) according to UGT1A1*1/*1, UGT1A1*1/*28 and UGT1A1*28/*28.

When considering the *UGT1A1* allelic status, the median concentration (IQR) of [RAL]_plasma_ was 88.5 ng/ml (41.0–236), 168 ng/ml (85.8–318) and 92.5 ng/ml (36.4–316) for UGT1A1*1/*1, UGT1A1*28 carriers and UGT1A1*36 carriers, respectively (p = 0.032) with a statistically significant difference between UGT1A1*1/*1 and UGT1A1*28 carriers (p = 0.030) (Table 3, Fig. 2).

**Table 3:** RAL plasmatic concentrations [RAL]_plasma_ and [RAL-glu]_plasma_/[RAL]_plasma_ ratio depending on *UGT1A1* allelic status.

	[RAL]_plasma_ (ng/ml)*	p-value		MR	p-value	
UGT1A1*1/*1 n = 42	88.5 (41.0–236)	0.03^**^	0.03^***^	5.8 (3–10)	0.006^**^	0.005^***^
UGT1A1*28 carrier n = 48	168 (85.8–318)			2.9 (1.6–5.3)		
UGT1A1*36 carrier n = 6	92.5 (36.4–316)			3.2 (1.7–5.9)		

^*^Concentrations are expressed as median with interquartile range (IQR).

^**^Kruskal-Wallis test.

^***^Steel–Dwass *post-hoc* test.

MR:metabolic ratio ([RAL-glu]_plasma_/[RAL]_plasma_).

The median MR (IQR) was 5.8 (3–10), 2.9 (1.6–5.3) and 3.2 (1.7–5.9) for UGT1A1*1/*1, UGT1A1*28 carriers and UGT1A1*36 carriers, respectively (p = 0.006) also with a statistically significant difference between UGT1A1*1/*1 and UGT1A1*28 carriers (p = 0.005) (Table 3, Fig. 2).

In relation to UGT1A1*28 polymorphism, the median concentrations (IQR) of [RAL]plasma were 88.5 ng/ml (41.0–236), 185 ng/ml (83.1–348) and 166 ng/ml (95–316) for UGT1A1*1/*1, UGT1A1*1/*28 and UGT1A1*28/*28, respectively (p = 0.039). However, when performing Steel-Dwass pairwise testing, none of the isolated paired association was significant as all associated p-values were > 0.05, with p = 0.12 between UGT1A1*1/*1 and UGT1A1*28/*28, p = 0.99 between UGT1A1*1/*28 and UGT1A1*28/*28 and p = 0.08 between UGT1A1*1/*1 and UGT1A1*1/*28. However, a slight linear trend across the different groups was observed when performing a parametric a priori polynomial linear contrast test (p = 0.022). The median MR (IQR) was 5.8 (3–10), 3.1 (1.4–5.9) and 2.6 (1.9–5) for UGT1A1*1/*1, UGT1A1*1/*28 and UGT1A1*28/*28, respectively (p = 0.007) with a significant difference between UGT1A1*1/*1 and UGT1A1*1/*28 or UGT1A1*28/*28 (p = 0.03 and p = 0.02, respectively) but not between UGT1A1*1/*28 and UGT1A1*28/*28 (p = 0.93). As for the [RAL]plasma, the results of the *a priori* linear polynomial contrast test confirmed a significant linear trend in the MR in function of the number of mutated alleles (*1/*1 > *1/*28 > *28/*28), suggesting an allele “dose-dependent” effect (Fig. 2, p = 0.02).

Values are reported on the Y-axis using a box and whisker plot. Bottom and top of the boxes indicate the 25th and 75th percentiles, respectively and the inside-line represents the median. Whiskers show maximal and minimal observed values.

## Discussion

We were able to show that the UGT1A1*28 defective allele has a significant impact on RAL exposure with higher [RAL]_plasma_ and lower MR among UGT1A1*28 carriers compared to UGT1A1*1/*1. This effect appeared allele-dose dependent: UGT1A1*28 homozygous having lower ratio than heterozygous carriers who in turn show lower ratio compared to wild-type homozygous. To the best of our knowledge, this is the first study reporting such a significant correlation in a cohort of HIV-1 infected patients originating from mixed ethnicities, particularly Caucasian and African.

The first study (case-control design) investigated the possible impact of genetic UGT1A1 defect on RAL exposure included 57 healthy subjects^17^. In their study, Wenning *et al*. demonstrated that carriership of the UGT1A1*28/*28 genotype (n = 30) was associated with a modest increase in RAL plasma exposure when compared to UGT1A1*1/*1 patients (n = 27). Although they did not observe any significant difference in AUC and C_max_ in UGT1A1*28/*28 patients compared to UGT1A1*1/*1 subjects (41, 40% higher respectively), these authors observed that UGT1A1*28/*28 subjects had a 91% higher value for concentration at the 12 h time point (C_12h_) as compared with UGT1A1*/*1 subjects. However, as acknowledge by the authors, there was a large degree of overlap in PK parameter values between the two groups. However, even if it minimizes the relative importance of the observed inter-group difference in C_12h_ when compared to the intra-group variability, this observation is in accordance with our results. Later, in a smaller study including 19 healthy subjects mostly Caucasian, Neely *et al*. did not find any influence of the UGT1A1*28 variant neither on RAL plasma exposure nor on the degree of RAL glucuronidation^18^. However, in this small cohort of patients, only one volunteer was homozygous for the UGT1A1*28 allele. Moreover, the first aim of this study was to compare in a crossover design the RAL plasma exposure between the standard dosage of RAL (400 mg BID) and a non-usual dosage (400 mg QD) combined with unboosted atazanavir (ATV) (400 mg QD), a protease inhibitor that inhibits UGT1A1. Consequently, their results are not comparable to ours as the pharmacokinetic parameters observed with the once-daily RAL combined with ATV are not equivalent to those of the current recommended twice-daily regimen as used in our study.

Among the studies with HIV-1 infected patients, Siccardi *et al*. did not show any significant correlation between UGT1A1*28 polymorphism and RAL trough plasma concentration with median values of 245 ng/ml (132–719), 261 ng/ml (91–515) and 212 ng/ml (95–1338) for patients homozygous UGT1A1*1/*1 (n = 36), heterozygous UGT1A1*1/*28 (n = 40) and homozygous UGT1A1*28/*28 (n = 10), respectively (p = 0.79)^19^. The participants of this study came from Italy without any precision about their ethnic origins. As the authors did not determine RAL-glu concentration, they were not able to compute the MR, a direct indicator of UGT1A1 activity towards RAL, in contrast to our study.

Later, Hirano *et al*. analyzed the *UGT1A1* genotype (including *6 and *28 determination) in a cohort of 56 Japanese HIV-infected patients treated with RAL^16^. UGT1A1*6 and UGT1A1*28 variants were found in 15 (among which two homozygous UGT1A1*6/*6) and 11 patients (with no homozygous UGT1A1*28/*28), respectively. Patients heterozygous for either the UGT1A1*6 or the UGT1A1*28 allele did not show different plasma RAL concentrations when compared to the wild-type homozygous (p = 0.23 and p = 0.50, respectively). Importantly, they stressed that both homozygous UGT1A1*6/*6 patients showed contradictory results. Indeed, they observed that one patient had low RAL plasma concentration while the other had high RAL plasma concentration when compared to patients homozygous for the with wild-type allele. It must be noted that, in the study of Hirano *et al*., there were no homozygous UGT1A1*28/*28 patients and neither the RAL-glu concentration nor the metabolite ratio was investigated.

Finally, Yagura *et al*. compared the effect of UGT1A1*6 and UGT1A1*28 variants on plasma RAL concentrations in a cohort of 114 Japanese HIV-infected patients^14^. In their study, the allelic frequencies for UGT1A1*6 and UGT1A1*28 were 18 and 13%, respectively. RAL plasma through concentrations were significantly higher in patients homozygous for UGT1A1*6 allele (n = 7) compared to patients UGT1A1*1/*1 (n = 56), with median value of 1000 ng/ml and 110 ng/ml, respectively (p = 0.021). When all genotype combinations were considered, the authors observed slightly higher RAL exposure among patients homozygous for one of the defective alleles (*28 or *6) when compared to UGT1A1*1/*1 patients with heterozygote carriers showing intermediate values. Indeed, UGT1A1*28/*28 (n = 4) and UGT1A1*6/*28 (n = 2) patients had median RAL plasma through concentrations of 280 and 290 ng/ml, respectively, while patients carrying one UGT1A1*6 (n = 25) or UGT1A1*28 (n = 20) defective allele showed values of 200 or 150 ng/ml, respectively. However, these differences were not statistically significant when compared to the median RAL concentration measured in homozygous wild-type UGT1A1*1/*1. Subsequently, the authors analyzed factors associated with high RAL plasma concentrations (defined as ≥170 ng/ml, the median RAL plasma trough concentration) using multivariate logistic regression: the presence of at least one UGT1A1*6 allele or two UGT1A1*28 alleles were considered as independent factors predicting high RAL plasma concentration. The authors demonstrated that the effect of UGT1A1*6 was dominant whereas their data suggested that the UGT1A1*28 defect was recessive. Again, this study did not investigate the RAL glucuronoconjugation rate.

In conclusion, most of the previous studies failed to demonstrate a correlation between RAL plasma exposure and UGT1A1*28 polymorphism. RAL is well known to have erratic pharmacokinetic profile with high intra- and inter- individual variability and coefficients of variations (CV) of 122–245% and 110–212% when considering the plasma concentrations within the same individual or between individuals, respectively^23,24^. This high degree of variability observed in the PK behavior of RAL combined with the small sample size of the above-mentioned cohorts^16–18^ has a potential negative impact on the statistical power and might explain why the UGT1A1*28 allele is not always significantly associated with variations of RAL plasma exposure by hiding the true pharmacogenomic effect of this variant. Moreover, two of the three studies involving HIV-1 infected patients were conducted on Japanese patients with no or few patients carrying UGT1A1*28 allele^14,16^ unlike our study involving 48 (50%) UGT1A1*28 carriers, probably explaining why, contrarily to us, they were not able to highlight the metabolic defect caused by the UGT1A1*28 allele. Lastly, in the only study involving Caucasian HIV-infected patients showing no impact of UGT1A1*28 polymorphism on RAL exposure, no information was provided about RAL metabolic ratio^19^. However, in our opinion, MR is probably the best indicator of UGT1A1 activity towards RAL because it better reflects the enzyme activity than the concentrations of the parent drug.

In relation to the clinical impact of our observation, it is important to stress that, even in naïve patient, a significant correlation was found between low RAL plasma trough concentration and the risk of virological failure^25^, particularly in the presence of a high viral load at baseline when RAL was administrated in once-daily regimen instead of twice daily and or in some NRTI-sparing regimen^26–28^. Furthermore, virological failure have been reported in treatment experienced patients when RAL was used to replace high genetic barrier drug^29,30^. Consequently, our study emphasized that RAL therapy can be improved through the screening of UGT1A1*28 allele, particularly when risk factors for virological failure are present: high viral load at baseline, once daily regimen or when RAL is used to replace high genetic barrier drug in treatment- experienced patients.

Consistent with the fact that RAL is generally considered as a well-tolerated drug, we observed very few clinical AE or biological abnormalities^2,3,24^ and, with the exception of the sentiment of fatigue that is an unspecific AE, none of our reported AE or biological abnormalities was correlated with either *UGT1A1* polymorphisms or RAL exposure (Fig. 1). The increased risk of fatigue we observed suggests that, if this association is real, the risk of suffering from this side effect is related to a higher metabolic conversion of RAL into its glucuronide. To our knowledge, this association has never been described previously and consequently, must be interpreted with prudence. Moreover, in our cohort, we observed an uncommon higher proportion of patients complaining of fatigue compared with what was previously reported^3^. One possible reason for explaining those discrepancies is the difficulty of relating this nonspecific symptom directly to RAL, as it could have many different etiologies. This might inexorably lead to spurious/confused associations, potentially explaining why we found a significant link between this AE and the MR, especially considering the borderline significance of this association (p = 0.048).

In some studies, elevated CPK have been reported after RAL administration, reaching up to 21% of the patients^31^. In our study, only one patient complained of muscular pain associated with significant elevation of CPK (grade 2). This patient was an African woman aged of 48 years, who had been treated by RAL for 51 months at day of inclusion. She was homozygous for UGT1A1*1 allele with [RAL]_plasma_ at 1312 ng/ml. The CPK level remained elevated even after RAL was changed in favor of dolutegravir, 20 months after the day of inclusion into the study. As a consequence, it is not likely that CPK elevation was due to RAL overexposure. Concerning the only case of grade 2 hyperbilirubinemia (>1.5–3.0 × ULN), it corresponds to a patient UGT1A1*28/*28 homozygous receiving ATV (and RAL) in combination. As both ATV and the UGT1A1*28/*28 genotype are associated with a decreased function of UGT1A1, this probably explains this observation. In line with this, Kozal *et al*. reported a higher number of cases of severe hyperbilirubinemia when unboosted ATV (300 mg BID) was co-administered with RAL (400 mg BID) compared to ATV 300 mg boosted with low doses of ritonavir (RTV) in combination with tenofovir 300 mg/emtricitabine 200 mg^32^.

Our study does, however, have some limitations. To our knowledge, the present study constitutes the largest cohort of mixed ethnicities RAL-treated HIV patients, but it still remains a relatively small number of patients to ensure maximal statistical power. This could potentially explain why we only observed a PK effect but no association between *UGT1A1* genotypes and clinical outcomes. Furthermore, in term of PK investigation, we only obtained information about plasma exposure through the measurement of trough concentration (C_min_). This point quantification cannot be considered as a perfect surrogate of the total RAL plasma exposure, such as AUC_0–12h_. However, C_min_ remains the most practicable dosage implementable in daily clinical practice and is still considered as valuable marker for therapeutic drug monitoring purpose^33^.

## Conclusions

To the best of our knowledge, this is the first report including such a large cohort of Caucasians and African HIV-1 infected patients that demonstrated a significant impact of UGT1A1*28 variant on RAL exposure with UGT1A1*28 carriers showing higher [RAL]_plasma_ and lower MR when compared to UGT1A1*1/*1 and this effect appeared to be allele-dose dependent. Except for the sensation of fatigue, this PK effect did not correlate with any clinical adverse events or biological abnormalities.

As some virological failures have been associated with low RAL exposure, UGT1A1*28 genotyping may still be considered as an interesting tool to improve RAL therapy particularly when risk factors for virological failure are present: high viral load at baseline, once daily regimen or when RAL is used to replace high genetic barrier drug in treatment- experienced patients. Further clinical studies are required to confirm this hypothesis.
